# Atmospheric Deposition of Polycyclic Aromatic Hydrocarbons (PAHs) in the Coastal Urban Environment of Poland: Sources and Transport Patterns

**DOI:** 10.3390/ijerph192114183

**Published:** 2022-10-30

**Authors:** Patrycja Siudek

**Affiliations:** Institute of Meteorology and Water Management, Waszyngtona 42, PL-81-342 Gdynia, Poland; patrycja.siudek@imgw.pl

**Keywords:** PAHs, deposition, seasonal distribution, sources, PM_2.5_, atmospheric transport

## Abstract

This study combines an interseasonal variation of deposition profiles of fine-particulate-bound polycyclic aromatic hydrocarbons (PM_2.5_-bound PAHs) with source apportionment analysis. Comprehensive measurements were conducted in four representative periods of 2019 in the coastal urban region of the Baltic Sea in Poland. The mean daily deposition flux of Σ_13_PAHs was 229 ng m^−2^ day^−1^, which was lower than in other urban/industrial sites of Europe and Asia. The seasonal PAHs distribution exhibited a clear U-shaped pattern, reaching maximum values in January and December and the minimum in June. A strong influence of local/regional anthropogenic emissions and meteorological factors (precipitation, ambient temperature, wind regimes) was observed. The contribution of medium molecular weight PAHs (fluoranthene, pyrene, benzo(a)anthracene, chrysene) to the total sum of PAHs deposition fluxes increased from 24% in spring to 38% in summer, as a result of photochemistry, urban traffic, and shipping emissions. The highest contribution of 5- and 6-ring PAHs occurred primarily in autumn (55%), followed by winter (39%), spring (35%), and summer (26%). Benzo(a)pyrene (human carcinogenic compound) had a relatively high deposition flux in winter, which was almost 14 and 20 times higher than the values registered in spring and summer, respectively. The FLEXTRA dispersion model was used to study potential pollution regions for PM_2.5_-bound PAHs and to investigate changes in the PAH deposition regime in different seasons. This study reveals that the winter contribution of PAHs was mostly impacted by local urban activities (i.e., residential heating and coal-fired power plants). Winter PAH deposition fluxes were particularly associated with atmospheric particles transported from surrounding areas and industrially impacted regions of SE–S–SW Poland and Europe.

## 1. Introduction

Polycyclic aromatic hydrocarbons (PAHs) are well-known, environmentally persistent pollutants with toxic and mutagenic properties [1]. PAHs can be largely emitted from different stationary sources such as industrial sites, smelters, and power stations during different processes such as incomplete pyrolysis of organic matter, thermal processing of food, biomass/waste combustion, and coal combustion in domestic heating [2,3,4]. Other large sources of atmospheric PAHs are wildfire biomass burning [5] and vehicle exhausts [6]. In coastal regions, the potential anthropogenic origins of PAHs also include port and shipbuilding activities, oil spills, and sea transport. In addition, the semi-volatile PAH compounds can be naturally emitted from the sea surface.

The USEPA has distinguished 16 priority native polycyclic aromatic hydrocarbons, including the compounds consisting of two rings: naphthalene (Naph); three rings: acenaphthylene (Acy), acenaphthene (Ace), anthracene (Ant), phenanthrene (Phe), fluorene (Flu); four rings: fluoranthene (Flt), chrysene (Chry), benzo(a)anthracene (BaA), pyrene (Pyr); five rings: benzo(b)fluoranthene (BbF), benzo(k)fluoranthene (BkF), benzo(a)pyrene (BaP), dibenzo(a,h)anthracene (DahA), and six rings: benzo(g,h,i)perylene (BghiP), indene (1,2,3-cd) pyrene (IcdP). These compounds differ from one another mainly in molecular structure, which determines their volatility, reactivity and toxicity as well as the rate of photochemical degradation. It is obvious that urban emission has an impact on PAH atmospheric chemistry, air quality, climate change, human health, and environmental conditions. According to the IARC classification, benzo(a)pyrene (BaP) belongs to the first group of carcinogenic substances and can be identified as a key marker of PAH toxicity in many environmental matrices. The European air quality standard for BaP is established at 1 ng per m^3^ (annual target value). However, in many urban agglomerations, this threshold is still regularly exceeded [3,7]. Therefore, comprehensive investigations of atmospherically deposited carcinogenic and mutagenic substances are particularly important in the context of climate change [8].

Deposition processes represent some important mechanisms of PAH removal from the atmosphere to the Earth’s surface and play a key role in their biogeochemical cycling in the environment, on local, regional, and global scales [9,10]. Deposition fluxes of PAHs are temporally and spatially variable, being the result of a large variation in PAH loadings over different regions and time periods [11,12]. It has been indicated that the size-dependent deposition velocity of semi-volatile compounds can vary greatly, mainly due to the fact that this parameter is closely linked to meteorological conditions, properties of the receptor surface, aerosol hygroscopicity and chemical composition [13]. Nevertheless, in many environmental studies, it is a priori assumed that the size distribution of particles and content of toxic compounds in the airborne PM remains constant during the study period. For example, Tasdemir and Esen [13] have provided a seasonal analysis for the deposition of PAHs adsorbed onto atmospheric particles in a residential area in Bursa, Turkey. Their calculations included deposition velocity of 0.5 cm s^−1^ and clearly showed considerable variability of PAHs distribution.

In recent years, several studies have examined deposition fluxes of PAHs in urban environments [10,14,15,16,17]. It was reported that deposition fluxes of PAH isomers (i.e., Phe, Flt, BbF, BkF, and IcdP) tend to be higher during the cold season due to high concentrations of medium- and high-molecular-weight PAH isomers. Most of the studies highlighted that the seasonal variability of PAH deposition patterns has a fundamental meaning for a better understanding of their cycle in the atmosphere. However, direct and comprehensive PAH measurements are still lacking in many coastal regions. For example, the relationships between PAH deposition patterns and individual meteorological factors (i.e., wind speed, humidity, air temperature fluctuations, precipitation) in coastal locations have not been investigated. So far, interseasonal and interannual variations in deposition fluxes of three-ring to six-ring PAHs have not been determined for the coastal areas in northern Europe.

This is the first comprehensive study dealing with deposition fluxes of atmospheric top priority PAHs (as defined by USEPA) in the coastal urban region of the southern Baltic Sea. This work presents the results of a 1-year project focused on PM_2.5_ measurements. The main objectives of this study were as follows: (1) to investigate profiles of dry deposition fluxes of low-, medium- and high-molecular-weight PAHs in four seasons; (2) to determine the transport pathways of PAHs, from their sources to a receptor site, using a standard backward trajectory model; (3) to analyze PAHs deposition fluxes in relation to different wind regimes; and (4) to examine relationships between PAHs concentrations/deposition and precipitation.

## 2. Materials and Methods

### 2.1. Details of Field Measurement Strategy

Fine particulate matter samples with a diameter of less than 2.5 µm (PM_2.5_) were collected from January to December 2019, in Gdynia (54°52′55.1″  N, 18°56′13.5″  E). Gdynia is a typical city in the Pomorskie Province, with up to 250 000 inhabitants and an area of 135.14 km^2^. In Gdynia, there is one of the biggest international harbors of the southern Baltic Sea, with a shipbuilding industry (almost 3700 incoming ships in 2016), various local urban sources (i.e., high traffic roads, domestic and commercial sectors), and industrial emissions sites such as coal-fired power plants, municipal solid waste recycling units, petrochemical plants, and refineries.

The sampling site (SP) was located in a coastal urban area, as shown in Figure S1 of the Supplemental Information. In brief, a temperature-controlled automatic sampler was installed on the rooftop of the aquarium building (20 m above ground level and less than 100 m from the shoreline). The area is clear of any obstacles, buildings and trees as recommended by European regulations for aerosol sampling. The PM_2.5_ samples were collected by PNS-18T Comde Derenda (GmbH, Stahnsdorf, Germany) on pre-fired quartz fiber filters (QMA, diameter 47 mm, Whatman, UK) at a flow rate of 2.30 m^3^ h^−1^. The mean PM_2.5_ sampling time was 23 h and 55 min. A total of 349 filter samples were obtained.

Local weather conditions, including ambient temperature, relative humidity, atmospheric pressure and precipitation amount were registered using relevant meteorological devices, and the relevant monthly data are presented in Table S1 of the Supplementary Materials. In Gdynia, mean ambient temperature in 2019 ranged from −2.0 °C (January) to 20.5 °C (July). The relative humidity varied between 45% (June) and 98% (January), with a mean value of 78%. The lowest daily value of atmospheric pressure was 980 hPa (January), and the highest was 1048 hPa (February). The wind speed and direction were very variable parameters during the whole observation period and are discussed in the following section. In general, southerly winds were much more frequent during winter months, while strong westerly and southwesterly winds were dominant during spring and summer. Moreover, coastal winds from the N to E sectors were registered during spring and summer observations, while haze and fog events occurred most frequently during the spring months.

### 2.2. PM_2.5_-Bound PAHs Pretreatment and Analysis

Each collected sample was wrapped in aluminum foil, conditioned for 24 h in a glass desiccator, weighted and then folded again in foil and kept in a zipped bag in a freezer at −20 °C until further analysis. In this study, PM_2.5_ samples were extracted using an Accelerated Solvent Extractor (ASE350 model, Dionex, UK). The extraction was performed with dichloromethane (DCM) and *n*-hexane (Merck) in the ratio of 1:1 as a basic solvent. Briefly, 15 mL of mixture DCM/*n*-hexane was added to a tightly-capped ASE cell containing 1/4 of a filter sample. The extraction procedure was previously described by Siudek and Ruczyńska [18]. Prior to analysis, extracts were concentrated to about 1 mL in a rotatory evaporator under vacuum at 35 °C, and residues were then transferred to a pre-baked glass column filled with glass wool, 1 cm of anhydrous sodium sulfate (Na_2_SO_4_), 2 g of 2% activated silica gel, and 1 cm of anhydrous Na_2_SO_4_ on top and eluted with 20 mL of DCM and hexane solution (1:1, *v*/*v*) to the 100 mL heart-shaped flasks. The extracts were again concentered in a rotary evaporator to 1 mL, then acetonitrile (4 mL) and dichloromethane (4 mL) were added to the concentrates and again gently evaporated to 0.5 mL under a nitrogen stream at 35 °C. The final extracts were transferred into glass chromatographic vials and stored in a refrigerator.

The 13 USEPA priority PAHs were analyzed in aerosol samples by high-performance liquid chromatography coupled with fluorescence detection (HPLC-FLD). A Shimadzu modular Prominence HPLC system (Japan) was used, with Kinetex LC column (150 mm × 4.6 mm i.d., particle size of 3.5 μm, Phenomenex, Torrance, CA, USA), 50:50 solution of HPLC grade acetonitrile, and water as mobile phase at a flow rate of 0.5 mL min^−1^ and injection volume of 25 μL. The excitation and emission wavelengths for all target PAH isomers were as follows: Nap–Phe: excitation Ex, λ = 270 nm, emission Em, λ = 350 nm; Flu–Pyr: Ex, λ = 250 nm, Em, λ = 420 nm; BaA–Chry: λ = 270 nm, Em, λ = 390 nm; BbF–DahA: λ = 290 nm, Em, λ = 430 nm, BghiP–IcdP: λ = 360 nm, Em, λ = 460 nm. The obtained data were processed through the use of Shimadzu Image software version 5.85.

To check for potential contamination of quartz filters during the field campaign (sampling, transport, pre-treatment procedure) and to evaluate analytical precision and bias, field and procedural blanks were measured following the same procedure as for aerosol samples. The results obtained for blanks showed relatively low contamination, mostly within ±7%. Detection limits for PAHs expressed as three times the laboratory blanks ranged from 0.001 ng m^−3^ (benzo(a)anthracene) to 0.016 ng m^−3^ (dibenzo(a,h)anthracene). A certified reference material ERM-CZ120 in replicates (N = 10) was used for quality control of PAH measurements. The mean recoveries of individual PAH congeners were between 82 and 110% (blank filters loaded with 0.5 mg of CRM for PAH quantification). Note that PAH compounds such as naphthalene (Naph), acenaphthylene (Acy) and acenaphthene (Ace) due to high volatility and presence mainly in the gas phase were not considered in this study.

### 2.3. Calculation of PAHs Deposition Fluxes

In the present study, deposition fluxes of PM_2.5_-bound PAHs (**F*_dry_***) for each collected sample (ng m^−2^ day^−1^) were calculated using the following equation:

**F*_dry_*** = **C*_PAHi_*** × **V*_d_*** (1)

where: **C*_PAHi_*** is the i-analyte concentration (ng m^−3^) in a PM_2.5_ sample and **V*_d_*** is the deposition velocity of atmospheric particles during a sampling day (m s^−1^). It was assumed a priori that **V*_d_*** was 0.5 cm s^−1^ for fine particles. The same method for the quantitative dry deposition algorithm can be found in [19,20]. The monthly deposition fluxes of PAH congeners were obtained by summing up the daily values. The results are discussed in the next section.

### 2.4. FLEXTRA Model Application

Meteorological simulations are crucial to better characterize variation of air mass transport and particulate pollutants deposition pathways within the study domain. An important kinematic option in atmospheric studies is a three-dimensional FLEXTRA model (NILU and Institute of Meteorology and Geophysics, Vienna, Austria). This air-mass transport model is based on numerical meteorological data provided by European Centre for Medium-Range Weather Forecast (ECMWF) and is used to calculate and present backward trajectory simulations. A detailed technical description of the model setup can be found elsewhere [21]. Here, the FLEXTRA product was used for studying the transport and origin of particles and to identify periods that were characterized by high deposition fluxes of PAHs (Figure S2 of the Supplementary Materials). The backward trajectory simulations (BTs) were computed for daily PM_2.5_ measurements. As a result, 349 cases were obtained. They were also simulated at three different arrival heights, i.e., 500, 1000, and 1500 m, to calculate boundary layer conditions. In addition, the results of PAH deposition fluxes associated with prevailing circulation patterns were divided into wind sectors, including N—north, NE—northeast, E—east, SE—southeast, S—south, SW—southwest, W—west, NW—northwest and L—local to facilitate the analysis of different local source contribution.

As can be seen in Figures S1 and S2, the N sector represents marine sources and relatively clean air masses, originating from northern European countries (i.e., Norway, Sweden, Finland), which passed over the Baltic Sea area. The NE sector covers plenty of docks and ports, with a large local coal-fired power plant. In the E sector, there is an area of the Gulf of Gdańsk coastal waters and the approach fairway to the international port of Gdynia. The SE sector is associated with southeasterly air mass advection coming from the emission regions in Ukraine and Russia. This sector is also linked to the second local coal-fired power plant in Gdańsk, the port and docks area, and a large complex of petrochemical refineries. The S sector includes major industrial sites in Poland (Upper Silesia region), and southern parts of Europe (i.e., Balkans, Italy, Croatia, Romania, Hungary, Czech Republic, Slovenia) with noticeable influences from the Mediterranean Sea region. The SW cluster includes local/regional traffic-related sources, residential heating, and industrial activities and also reflects the impact of polluted regions in southwestern Europe, including the Czech Republic, Austria, Switzerland, and Spain. The W sector represents the local road network, residential area, and municipal solid waste recycling units in the central and western European countries (i.e., France, Germany, Austria) with various industrial/urban activities. The NW cluster represents the flow of air from northwestern European areas (i.e., Ireland, Island, the United Kingdom, Denmark, the Danish Straits, northern Germany), the North Sea, and the Atlantic Ocean, thereby providing a mixture of maritime aerosol with trace gases and other pollutants.

To investigate the circulation pattern and transport of pollutants over the study domain, the cluster analysis of one-day FLEXTRA backward trajectory simulations was used. The results are discussed in Section 3.4.

## 3. Results

### 3.1. PAHs Deposition in Gdynia and Other Worldwide Sites

In the present study, the mean daily deposition flux of total PAHs was 229 ng m^−2^ day^−1^. The mean Σ_13_PAHs ± 1σ (min-max) deposition flux (in ng m^−2^ day^−1^) in spring, summer, autumn and winter was: 98 ± 86 (10.7–423.8); 47.2 ± 25.6 (15.9–165.4); 223.1 ± 168.3 (21.4–800.2), and 523.8 ± 483.1 (16.4–2278.6), respectively. The seasonality of PAH deposition was clear, suggesting a major role of residential heating, which occurs mostly in the winter season. This trend is consistent with several observations from previous PAH-oriented studies [11,22]. In Gdynia, deposition fluxes of PAHs were similar to the values registered in Kathmandu, Nepal (281 ng m^−2^ day^−1^ for Σ_15_PAHs) [23] and Mottola, Italy (211 ng m^−2^ day^−1^ for Σ_7_PAHs) [15]. However, the annual mean PAH deposition flux in this study was much lower than values observed in other urban and industrial areas of Europe [15] and Asia [17]. For instance, the annual deposition of Σ_7_PAHs was 1012 ng m^−2^ day^−1^ in Taranto, Italy [15], and slightly higher values were found along the continent in the Russian Arctic (1108 ng m^−2^ day^−1^ for Σ_35_PAHs) [24]. A high deposition for Σ_14_PAHs (3300 ng m^−2^ day^−1^) was observed in polluted regions of Bursa, Turkey [25]. Similar patterns of high values of PAHs deposition fluxes were identified in Shanghai and Beijing, where average PAH deposition fluxes were estimated at 4060 ng m^−2^ day^−1^ [17] and 5140 ng m^−2^ day^−1^ [26], respectively, indicating that PAH physical and chemical processes were largely influenced by industrial sources, residential heating activities and atmospheric conditions. Previous studies carried out in highly polluted urban environments have also reported remarkably high values of toxic semi-volatile compound deposition, reaching up to 35,000 ng PAHs per m^−2^ per day^−1^ [27]. 

### 3.2. Seasonal Variability of PAHs Deposition Fluxes

In this study, the daily minimum concentration of atmospheric fine particulate matter was observed in July (1.72 µg m^−3^), while the peak concentration of PM_2.5_ was reported in February (167 µg m^−3^). The mean annual PM_2.5_ concentration was 24.1 ± 22.5 µg m^−3^. In Gdynia, high concentrations (>50 µg m^−3^) of PM_2.5_ are commonly observed during the cold season (from December to March) due to intensive emissions in local residential (i.e., coal combustion, domestic heating units) and commercial sectors (coal-fired power plants).

**Figure 1:**
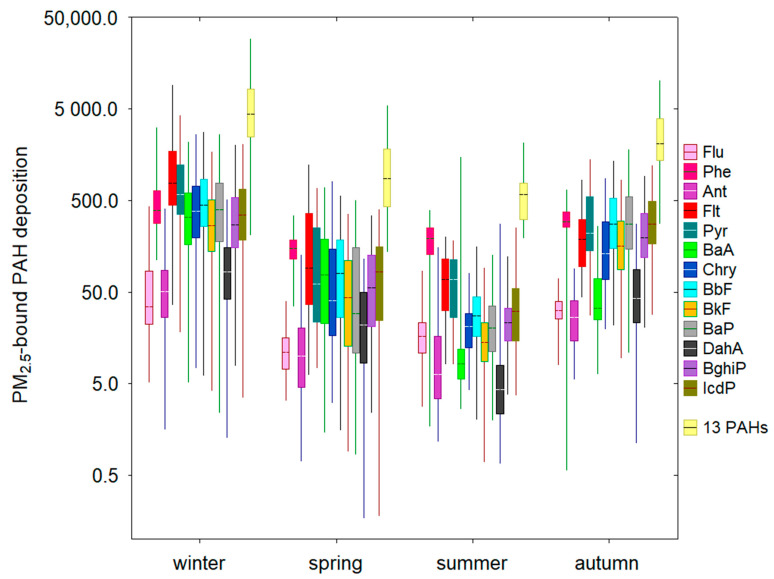
Seasonal boxplots of PM_2.5_-bound PAH deposition fluxes in the coastal urban region in northern Poland, 2019. Box edges denote the 25th and 75th quartiles, the median value is indicated as a vertical line inside boxes, and whiskers stand for minimum and maximum.

Atmospheric deposition of 13 individual PAH compounds exhibited large seasonal variation (Figure 1). In general, higher deposition fluxes of PAH isomers were found in winter (January/February/December), when local residential coal combustion is more intensive. The fluxes were significantly lower in summer. The median total deposition flux of Σ_13_PAHs was almost eight times higher in winter than in summer (Figure 1).

The highest median deposition flux was observed for Flt (770.2 ng m^−2^ day^−1^), followed by Pyr (582.5 ng m^−2^ day^−1^) and BbF (442.4 ng m^−2^ day^−1^). In winter, the median BaP deposition flux was 396.2 ng m^−2^ day^−1^, which was almost 14 and 20 times higher than the values registered for spring and summer, respectively. Similarly, median deposition fluxes for such PAHs as Phe (392.5), Chry (380.6), IcdP (347.8), and BaA (327.7) were much higher in winter as compared to the values in other seasons (Figure 1). In this study, the median deposition flux for highly toxic BghiP was 270.4 ng m^−2^ day^−1^ in winter, followed by autumn (196.9 ng m^−2^ day^−1^), spring (55.4 ng m^−2^ day^−1^), and summer (22.9 ng m^−2^ day^−1^), indicating the important influence of anthropogenic activities such as coal combustion, gasoline vehicle emission, and local mixed sources. The highest median value of BbF was also observed in winter, while 15 times lower deposition BbF levels occurred in summer (Figure 1). This observation can be partly explained by large differences in the emission sources (such as fossil fuel combustion for residential heating and traffic emission) for high-molecular-weight PAHs in the study area. BbF is a typical marker associated with traffic-related emissions [28]. In the present study, the main traffic emission sources of BbF were located NW to SE of the sampling site. As shown in Figure 1, median PAH deposition fluxes (ng m^−2^ day^−1^) in autumn were in the following order: Phe (292.9) > BaP (276.3) > BbF (274.8) > IcdP (273.4) > Pyr (221.2) > BghiP (196.6) > Flt (189.9) > BkF (157.2) > Chry (132.1) > DahA (42.8) > BaA (33.4) > Flu (31.5) > Ant (26.2). The relatively high deposition fluxes of BaP in relation to other six-ring PAHs could be related to industrial emissions.

### 3.3. Interseasonal Contributions to Deposition Fluxes

**Figure 2:**
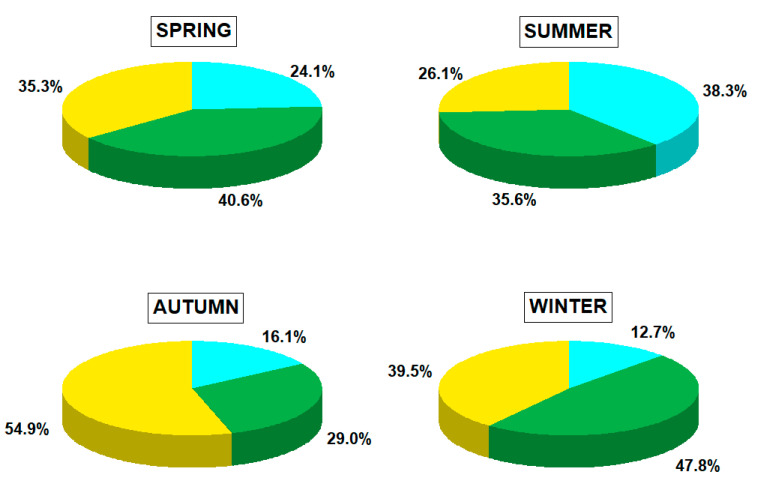
Pie-charts of low- (green), medium- (blue) and high-molecular-weight (yellow) PAH percentage share in total PAH deposition in four seasons.

Figure 2 shows the seasonal distribution of low- (166–178 g mol^−1^), medium- (202–228 g mol^−1^) and high-molecular-weight (252–278 g mol^−1^) PAHs atmospherically deposited in the coastal urban region of Poland. The contribution of PAHs in each category group to the total PAH deposition fluxes was significantly different (*p* < 0.05). Overall, the deposition of high-molecular-weight PAHs (i.e., BbF, BkF, BaP, DahA, BhgiP, IcdP) was predominant in autumn, followed by winter, spring, and summer. It accounted for 54.9%, 39.5%, 35.3%, and 26.1% of the total PAHs, respectively (Figure 2). Medium-molecular-weight PAHs (including compounds such as Flt, Pyr, BaA, and Chry) had a large contribution (47.8%) in winter, while in autumn their contribution to the total PAHs did not exceed 30.0%.

As shown in Figure 2, the contribution of low-molecular-weight PAHs (Flu, Phe, Ant) in winter was relatively small (12.7%). It was particularly evident for two- and three-ring PAHs, which exhibited low concentrations in the particle phase due to their high volatility [29]. Additionally, the effect of the increased contribution of medium-molecular-weight PAHs (up to 38.3% in total PAHs) was observed in summer, indicating a high influence of shipping emissions in the nearby port area. Cooper [30] has mentioned that ships using heavier residual oils, such as fuels, are likely to have higher exhaust emissions of PAHs (e.g., Ant, Flt). Additionally, it has been previously reported that the combustion of marine fuel oil can be associated with a relatively high concentration of more volatile PAHs [31,32]. Zhao et al. [33] have recently observed the influence of engine type, fuel, and operating conditions on local concentrations of PAHs emitted from an inland-river bulk freighter using marine diesel oil and an ocean-going passenger vessel using heavy fuel oil in the coastal urban region of China. They revealed that both ships showed a high proportion of four-ring PAHs (Flt, Pyr, BaA, Chry). Moreover, inland-river bulk freighters emitted more three-ring PAHs (34–70%), while ocean-going passenger vessels released more five-ring PAHs (30–36%). This study showed that concentrations of Σ_17_PAHs measured in ship exhausts ranged from 1.95 to 417 μg m^−3^ [33]. This is consistent with the seasonal trend of PAHs in the coastal urban region in Poland observed in this study. In particular, the trend from the warm study period when air pollution episodes can be significantly enhanced by northerly and easterly winds from marine areas suggests a large influence of shipping activities on local PAH concentrations and notable differences in deposition patterns. The contribution of ship emissions to atmospheric PAH distribution is discussed in the next section.

**Figure 3:**
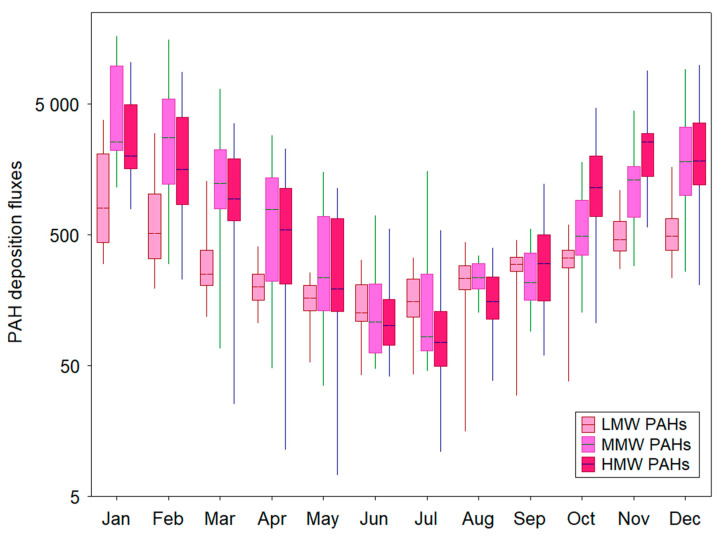
Monthly distribution of low-, medium- and high-molecular-weight PAH deposition fluxes in the coastal urban region of northern Poland. Box edges denote the 25th and 75th quartiles, the median value is indicated as a vertical line inside boxes, and whiskers stand for minimum and maximum.

Figure 3 provides the monthly distribution of low-molecular-weight (LMW: Flu, Phe, Ant), medium-molecular-weight (MMW: Flt, Pyr, BaA, Chry), and high-molecular-weight (HMW: BbF, BkF, BaP, DahA, BghiP, IcdP) PAHs in Gdynia. Specifically, deposition peaks were found for total medium-molecular-weight PAHs during the winter months (January and February: 16,594.8 and 15,745.7 ng m^−2^ day^−1^) and the lowest deposition fluxes were registered for total high-molecular-weight PAHs in May (7.4 ng m^−2^ day^−1^, Figure 3).

These results are in good agreement with other studies that revealed higher concentrations and deposition fluxes of medium- and high-molecular-weight PAHs in the coldest season due to considerably higher residential coal and wood combustion [34]. Other studies have also highlighted that meteorological conditions may favor faster accumulation and higher concentrations of airborne particles in winter [35]. Several atmospheric parameters are the most influential for the increasing trend of PAH concentrations in winter, i.e., low ambient temperature, weak solar radiation, reduced photochemical activity, and relatively low atmospheric mixing height [9,18,36]. In turn, the spring and summer periods are characterized by decreasing trends of the contribution of four- to six-ring PAH compounds to the total PAHs and also by an increasing trend of low-molecular-weight PAHs deposition. These observations suggest that different sources and processes, partly resulting from the variation in atmospheric conditions (i.e., low relative humidity, volatilization, higher temperatures, and photochemical activity), may remarkedly lead to changes in PM_2.5_ chemical composition and PAHs transformation in the coastal atmosphere during summer. A more recent study by Tsiodra et al. [37] has shown a very similar seasonal cycle of semi-volatile PAH compounds in Athens, Greece. In their study, high-molecular-weight PAHs were the most abundant PAH fraction in autumn and winter (51% and 60%, respectively), resulting from local/regional biomass burning processes. In contrast, low-molecular-weight PAHs were highest in summer (49%), reflecting the influence of enhanced photochemical processing. It is usually considered that low-molecular-weight PAHs are formed during low-temperature processes such as biomass burning [38]. This finding can be partly attributed to the impact of biomass burning within the Polish study domain, which was confirmed by the relatively high contribution of low-molecular-weight PAHs to total PAHs (Figure 2) and their seasonality (Figure 3).

### 3.4. Overview of PAHs Deposition Fluxes in Different Wind Regimes

**Figure 4:**
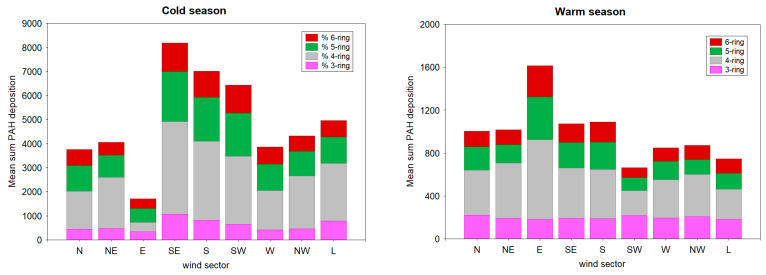
The cumulative mean sum of PAH deposition fluxes in relation to the wind sector during the cold and warm seasons.

The sources of PAHs were identified based on backward trajectory analysis. In brief, the main regional/distant pollution areas were examined in each cluster based on the FLEXTRA model simulations, as mentioned in Section 2.4. Differences in deposition fluxes obtained for each PAH group are displayed in Figure 4. The results were obtained for different seasons described as the cold and warm seasons.

Among the nine wind clusters, the highest mean Σ_13_PAHs (8180.8 ng m^−2^ day^−1^) was observed for the SE sector during the cold season, suggesting that local/regional anthropogenic sources had a major influence on the PAHs budget in the study area (Figure 4). For the SE cluster, deposition of PAHs (ng m^−2^ day^−1^) was ranked as follows: Σ3-ring PAHs (1060.5) < Σ6-ring (1191.5) < Σ5-ring (2074.9) < Σ4-ring (3854.0), suggesting a large contribution from coal-fired plants and the emission of liquid fossil fuels, including oil, gasoline, and diesel from motor vehicles. Low-molecular-weight PAHs are well-known as key tracers for petrogenic sources, i.e., petroleum refineries and petrochemical manufacturing [35]. In this study, the petrochemical refinery and plants located SE from the sampling site also contributed to the total PAH concentration. Their role was pronounced during the cold season (Figure 4).

Moreover, high mean Σ_13_PAHs (>5000 ng m^−2^ day^−1^) values were observed for S and SW sectors, which mostly represented urban-influenced areas. The major identified PAHs sources in S–SW sectors were related to residual energy consumption, including domestic heating, traffic-related emissions, and other industrial activities, i.e., municipal solid waste recycling units. Except for the E sector, the PAH deposition patterns for all other wind clusters exhibited the same trend during the cold season, i.e., 4-ring > 5-ring > 6-ring > 3-ring. Deposition fluxes calculated for air masses transported from the E direction were characterized by relatively low values of three-ring (336.1 ng m^−2^ day^−1^), four-ring (395.2 ng m^−2^ day^−1^), six-ring (397.3 ng m^−2^ day^−1^) and five-ring (579.5 ng m^−2^ day^−1^) PAHs. This finding can be interpreted as a minor contribution to maritime transport during the cold season in this region. Such results coincide with previous observations demonstrated that the combustion-related processes play a more important role in PAH emissions and deposition during the cold season (December to March) compared with the warm season [14,16,17,25,34,39]. The main reasons for high deposition fluxes of PAHs in winter are intensive anthropogenic activities (i.e., residential heating) and meteorological conditions (lower atmospheric mixing height, decreased potential of photolytic reactions in the atmosphere).

It is noteworthy that the opposite trend occurred in relation to PAH distribution for the E wind sector during the warm season, indicating that shipping activities were much more important for the PAH budget during spring and summer in the study region. For this period, deposition fluxes of four-, five- and six-ring PAHs were substantially higher (734.4; 399.9, and 287.5 ng m^−2^ day^−1^, respectively) in relation to sources from the E sector as compared to other local sources associated with the remaining wind sectors (Figure 4). Comparing the large variation in ΣPAHs deposition observed in all S clusters during the heating season, it was determined that the variability of dry deposition in these target wind sectors was relatively small and insignificant during the warm season (Figure 4). For example, the deposition of PAHs from the S, SE, and SW sectors was generally 6, 8, and 10 times lower, respectively, in the warm season, when the contribution of local emission (i.e., solid biofuel burning and fossil fuel combustion) is largely reduced in this region.

### 3.5. Impact of Precipitation on PM_2.5_-Bound PAHs: Concentrations and Deposition Fluxes

**Figure 5:**
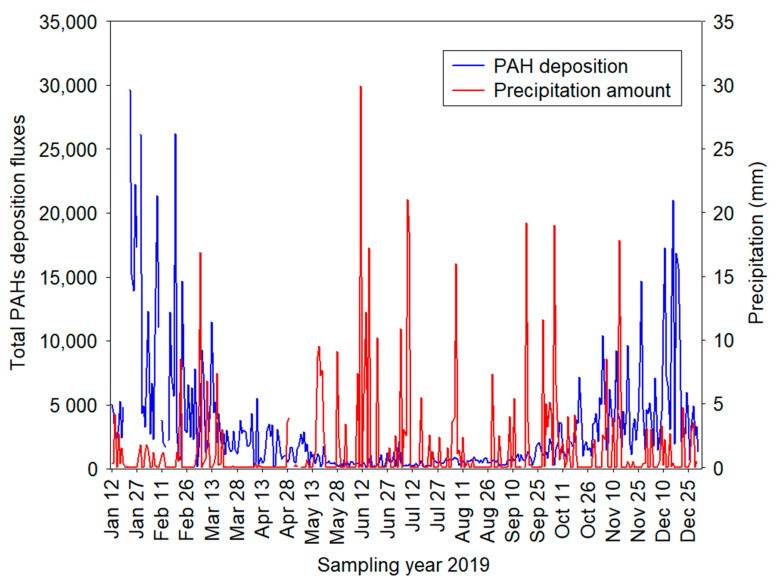
Daily profiles of ΣPAHs deposition fluxes (ng m^−2^ day^−1^) and precipitation (mm) during the study period 2019.

Figure 5 illustrates a time series of Σ_13_PAHs deposition vs. precipitation amount during the study period of 2019. The precipitation amount varied between 0.01 mm (January) and 29.9 mm (June), with a daily mean value of 1.48 mm.

It can be seen that the variability of total PAH deposition fluxes was substantially lower in summer compared with values measured in winter. It was mainly caused by: (a) reduced PAHs emission from local anthropogenic sources such as coal combustion and other high-temperature industrial processes; (b) high O_3_ concentration (mean: 66.1 mg m^−3^); and (c) higher precipitation amount (mean: 70.26 mm) and temperature (mean: 17.3 °C). This suggests some significant impact of direct photolysis, photochemical degradation, and reactions with atmospheric oxidants (e.g., OH radicals, NO_3_ radicals, O_3_, NO_2,_ Cl atoms) in the gas phase during summer. Keyte et al. [9] found that PAH lifetimes in terms of photochemical degradation range between 1 h and 14 days. Reisen and Arey [40] pointed out that PAH photolytic loss is more efficient in summer compared to winter, which is in line with the results of this study. Specifically, relatively low mean ΣPAHs dry deposition in summer months (June: 1060 ng m^−2^ day^−1^ < July: 1232 ng m^−2^ day^−1^ < August: 1565 ng m^−2^ day^−1^) coincided with a high concentration of O_3_ (June: 78.9 µg m^−3^ > August: 70.9 µg m^−3^ > July: 60.3 µg m^−3^) and relatively large precipitation amount (June: 88.22 mm > July: 72.23 mm > August: 38.73 mm), which may reflect a direct effect of atmospheric PAH loss, due to their photochemical degradation and below-cloud scavenging of PAH compounds associated with airborne PM. The dry deposition mechanism depends on the concentration levels and deposition velocities. As shown by Machado et al. [41], dry deposition may therefore be a major removal process for three-ring (Phe), four-ring (Flt), five-ring (BbF, BkF) and six-ring (IcdP) PAHs.

Substantially low monthly precipitation in April (total = 8.1 mm) possibly contributed to higher deposition fluxes (mean ΣPAHs = 4159 ng m^−2^) compared to May (mean ΣPAHs = 2385 ng m^−2^), when the total precipitation amount was 50.33 mm. As can be seen in Figure S3 of the Supplementary Materials, significant day-to-day variations were found for low-, medium- and high-molecular weight PAHs during the study period due to the effect of precipitation, regional transport of micropollutants, changes in meteorological conditions, as well as changes in emission rates of different sources. In the present study, differences between **F*_dry_*** PAHs and precipitation patterns were also evident throughout the winter measurements, when dry deposition of PAHs gradually increased (Figure 5). These observations can be also supported by Spearman’s rank correlation analysis between precipitation, PM_2.5_ loadings, and concentration of PAHs compounds (Table 1).

Several past studies recognized that precipitation has a negative influence on PM_2.5_ concentrations [42,43]. Specifically, episodes of heavy rainfall can have a strong washing-off effect on fine aerosols and may lead to a significant reduction of PM_2.5_ mass concentrations, while low precipitation may not remove PM_2.5_ effectively [43]. The authors have also mentioned that slight precipitation may induce enhanced relative humidity, and thus may lead to an increase in PM_2.5_ concentration. Considering PAH disposition fluxes, the correlation analysis between PAH concentrations and precipitation, examined separately for each sampling period, useful statistical information, and insight into the temporal variability of deposition profiles can be provided. In the current study, precipitation was negatively correlated to PM_2.5_ mass concentration (−0.470) and the following PAHs: BkF (−0.362), BbF (−0.358), BghiP (−0.355), Pyr (−0.346), Phe (−0.341), Chry (−0.327), BaP (−0.325), DahA (−0.318), Flu (−0.315), Flt (−0.305), IcdP (−0.303), BaA (−0.266), and Ant (−0.252) during the cold study period. In spring, the correlation coefficient between precipitation amount and PM_2.5_ mass concentration was weak and insignificant (R = 0.142, *p* < 0.05), while a significant negative and moderate correlation (−0.324 < R < −0.398, *p* < 0.05, Table 1) was revealed between precipitation and PAHs, excluding Flu, Ant, BaA, and DahA. The relatively weak correlations between precipitation and PAHs in summer (R from −0.089 to 0.121, *p* < 0.05) and autumn (R from −0.128 to 0.171, *p* < 0.05), in comparison with that in winter, suggest a substantially higher concentration of PAHs during the cold study period. In addition, the influence of precipitation on PAHs deposition variability may be smaller in summer (May to July) and autumn (September), as presented in Figure 2 and Table 1.

## 4. Conclusions

This study provides the first results from ground-based measurements of PAH deposition fluxes in the coastal urban region of northern Poland. Deposition fluxes of 13 PAHs varied between 10.7 and 2278.6, with a mean value of 229 ng m^−2^ day^−1^. They exhibited clear seasonal variation. The Flt, Pyr, BbF, and BaP deposition values were respectively 11, 8, 16, and 20 times higher in winter than in summer, suggesting that anthropogenic activities (i.e., coal combustion in residential/commercial sectors) were predominant sources of PAHs in winter and remarkably contributed to their high deposition fluxes. Based on FLEXTRA backward trajectory analysis, three wind sectors (SE, S, SW) were determined as potential source areas during the cold season. Specifically, the highest mean Σ_13_PAHs (8180.8 ng m^−2^ day^−1^) was observed for the SE sector, suggesting a large local impact of anthropogenic sources such as coal-fired power plants and petrochemical refineries on the PAHs budget. In contrast, during the warm season, the relative contribution of four-ring PAHs from the E sector increased almost two times in relation to the cold season, indicating that emissions from ship exhausts were relatively more important for the coastal PAHs budget in warm seasons.

This study indicates that PAH deposition data are important in controlling the seasonal emission profiles of various environmentally persistent pollutants. They provide reliable estimates of the future impacts of toxic PAHs on air quality and human health in the coastal urban region, as well as on global climate change.

## Figures and Tables

**Table 1:** Spearman’s rank correlation coefficients between precipitation and chemical variables (X: PM_2.5_ mass concentration/PAH concentrations). Statistically significant (*p* < 0.05) correlations are marked in red.

X	Spring	Summer	Autumn	Winter
	X vs. Precipitation	X vs. Precipitation	X vs. Precipitation	X vs. Precipitation
Flu	−0.108	0.046	0.171	−0.315
Phe	−0.396	0.020	0.069	−0.341
Ant	−0.106	0.090	−0.053	−0.252
Flt	−0.398	0.011	0.063	−0.305
Pyr	−0.368	−0.089	−0.128	−0.346
BaA	−0.040	0.121	−0.098	−0.266
Chry	−0.353	0.033	−0.028	−0.327
BbF	−0.360	0.076	−0.107	−0.358
BkF	−0.340	0.083	−0.089	−0.362
BaP	−0.324	−0.029	−0.111	−0.325
DahA	−0.150	0.090	−0.094	−0.318
BghiP	−0.332	0.058	−0.112	−0.355
IcdP	−0.375	0.065	−0.075	−0.303
LMW PAHs	−0.328	0.045	0.063	−0.342
MMW PAHs	−0.239	0.087	−0.049	−0.342
HMW PAHs	−0.323	0.100	−0.108	−0.347
PM_2.5_	0.142	−0.042	0.071	−0.470

## Data Availability

All data generated or analyzed during this study are included in this article (and its Supplementary Materials).

## Supplementary Materials

The following supporting information can be downloaded at: https://www.mdpi.com/article/10.3390/ijerph192114183/s1. Figure S1: PM_2.5_ sampling site in Gdynia, Poland; Figure S2: An example of 24-h backward trajectory simulation over the study domain; Figure S3: Daily variability of deposition fluxes of low-, medium-, and high-molecular-weight PAHs during the PM2.5 measurements in 2019; Table S1: Statistical parameters for PM_2.5_ mass concentration and meteorological variables in Gdynia, 2019.
