# Supplementary material for: Atmospheric Deposition of Polycyclic Aromatic Hydrocarbons (PAHs) in the Coastal Urban Environment of Poland: Sources and Transport Patterns
Source: Int J Environ Res Public Health. 2022 Oct 30;19(21):14183. doi: 10.3390/ijerph192114183 (PMC9657786; doi:10.3390/ijerph192114183)
Supplement: Supplementary file 1 [file ijerph-19-14183-s001.zip › ijerph-1990120-supplementary.pdf]

Supplement of

Atmospheric Deposition of Polycyclic Aromatic Hydrocarbons (PAHs) in the Coastal Urban Environment of Poland: Sources and Transport Patterns

*** Patrycja Siudek**

Correspondence to: *Patrycja Siudek (patrycja.siudek@imgw.pl)

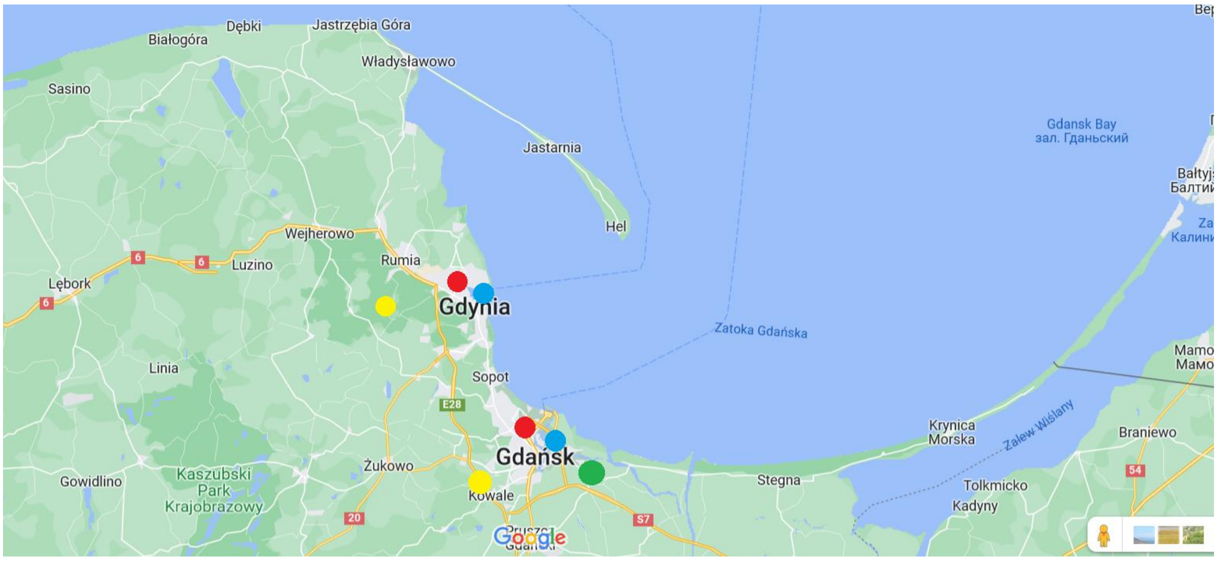

Figure S1. $PM_{2.5}$ sampling site in Gdynia, Poland. Dots represent major local/regional PAH sources, i.e. coal-fired power plant (red), port and docks area (blue), petrochemical refinery and plants (green), and municipal solid waste recycling units (yellow).

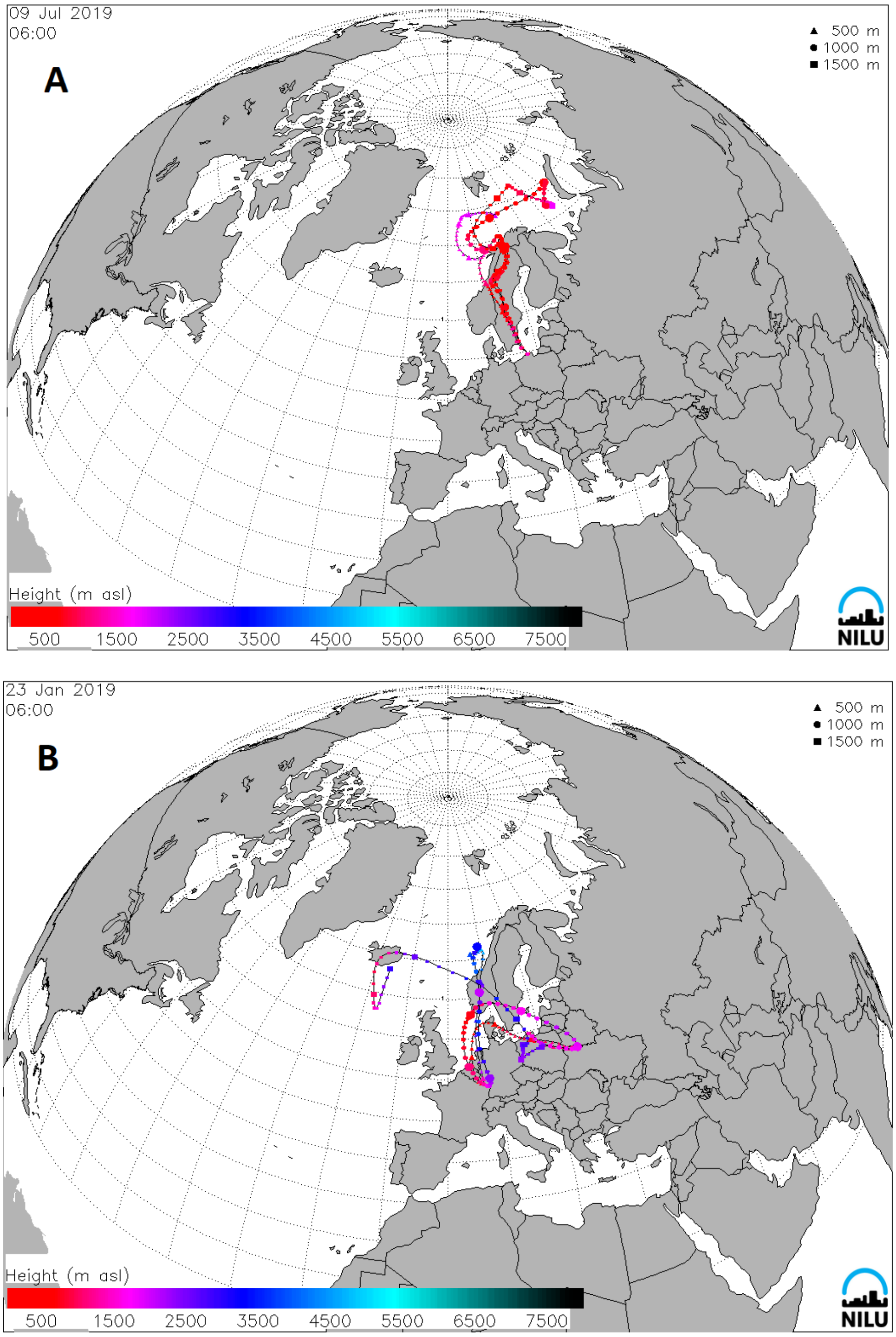

Figure S2. An example of 24-h backward trajectory simulation over the study domain. A chosen day with (**A**) low deposition fluxes (17.6 ng m^{-2} day^{-1}, precipitation amount of 21.0 mm) in the non-heating study period and (**B**) high deposition fluxes (2278 ng m^{-2} day^{-1}, precipitation amount of 0.01 mm) in the heating season. Results were obtained from the FLEXTRA model.

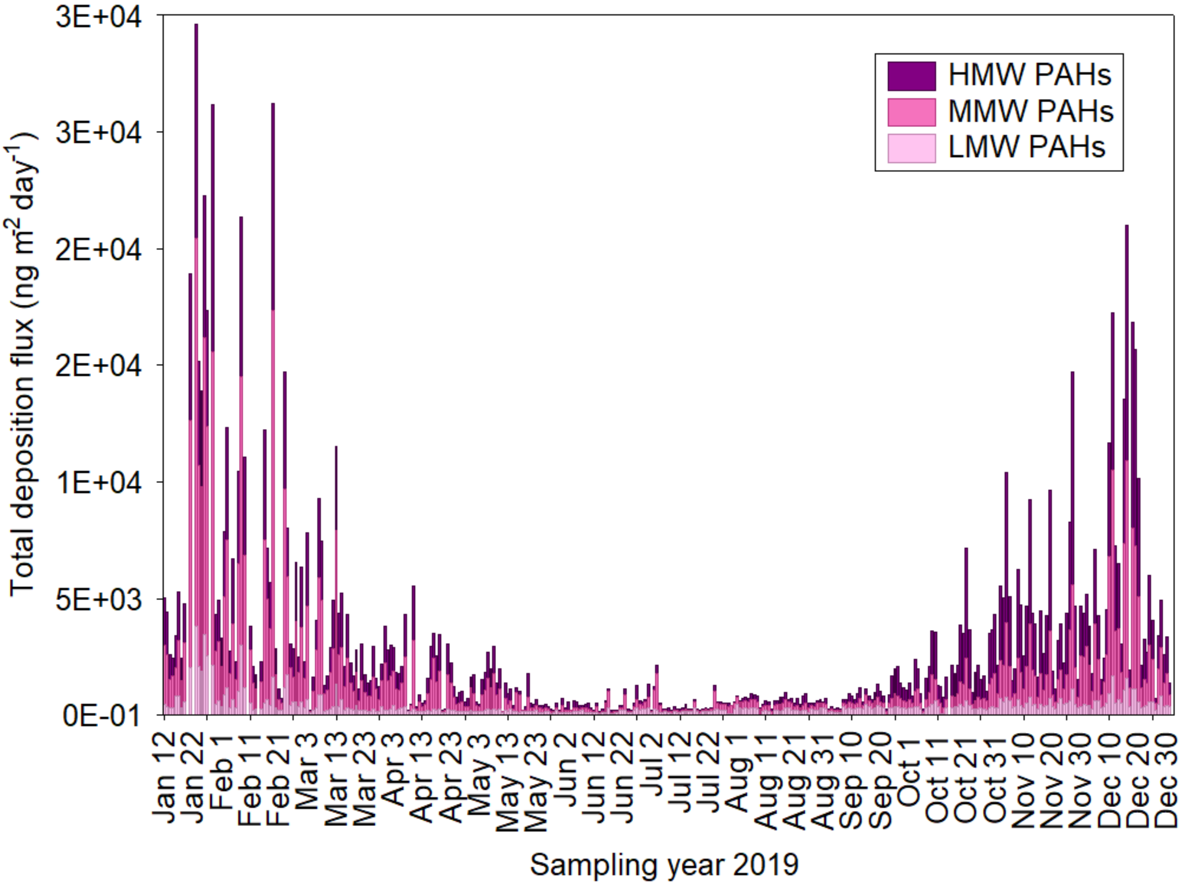

Figure S3. Daily variability of deposition fluxes of low-, medium-, and high-molecular-weight PAHs during the $PM_{2.5}$ measurements in 2019.

Table S1. Statistical parameters for $PM_{2.5}$ mass concentration and meteorological variables in Gdynia, 2019.

Meteorological parameter (unit)	Mean	SD	Min	Max
Air temperature (°C)	9.54	6.75	–6.60	25.20
Air pressure (hPa)	1011	10.06	980	1048
Relative humidity (%)	77.56	10.12	45.30	98.20
Precipitation height (mm)	1.49	3.67	0.01	29.9
$PM_{2.5}$ mass concentration (µg m^{-3})	24.26	22.52	167.24	1.72
